# Eight Years of Norovirus Surveillance in Urban Wastewater: Insights from Next-Generation

**DOI:** 10.3390/v17010130

**Published:** 2025-01-17

**Authors:** Giusy Bonanno Ferraro, David Brandtner, Pamela Mancini, Carolina Veneri, Marcello Iaconelli, Elisabetta Suffredini, Giuseppina La Rosa

**Affiliations:** 1 National Center for Water Safety (CeNSia), Istituto Superiore di Sanità, Viale Regina Elena 299, 00161 Rome, Italygiuseppina.larosa@iss.it (G.L.R.); 2 Department of Infectious Disease, Istituto Superiore di Sanità, Viale Regina Elena 299, 00161 Rome, Italy; 3 Department of Food Safety, Nutrition and Veterinary Public Health, Istituto Superiore di Sanità, Viale Regina Elena 299, 00161 Rome, Italy

**Keywords:** norovirus, wastewater, genotype, NGS

## Abstract

Human noroviruses (HNoVs) are a leading cause of acute gastroenteritis worldwide, with significant public health implications. In this study, wastewater-based epidemiology (WBE) was used to monitor the circulation and genetic diversity of HNoVs in Rome over an eight-year period (2017–2024). A total of 337 wastewater samples were analyzed using RT-nested PCR and next-generation sequencing (NGS) to identify genogroups GI and GII and their respective genotypes. The results showed that GII had higher detection rates (66.5%) compared to GI (50.7%), with significant variation between years. Detection rates peaked in 2019 before declining sharply in 2020, coinciding with the COVID-19 pandemic and rebounding after the pandemic in 2023. A total of 24 genotypes were identified (8 GI and 17 GII), including persistent variants GII.2, GII.3 and GII.4 and emerging genotypes such as GII.8, GII.10 and GII.14. Only two GII.4 variants, Sydney_2016 and Sydney_2012, were detected in the study. These results demonstrate the utility of WBE in tracking HNoVs circulation, identifying genotype diversity and capturing shifts in transmission dynamics. WBE provides a cost-effective and comprehensive tool for public health surveillance, particularly in regions with limited clinical surveillance. Sustained investment in WBE is crucial for advancing our understanding of HNoVs epidemiology and its long-term trends.

## 1. Introduction

Human noroviruses (HNoVs) are the leading cause of acute gastroenteritis cases worldwide, affecting individuals of all ages. These infections are estimated to cause 685 million cases of illness annually, including 200 million cases in children under 5 years of age and cause approximately 50,000 child deaths, mostly in developing countries (https://www.who.int/teams/immunization-vaccines-and-biologicals/diseases/norovirus) (accessed on 30 April 2022). The norovirus classification scheme was updated in 2019, based on VP1 and the RNA-dependent RNA polymerase (NS7) and includes 10 genogroups (GI-GX) and 49 genotypes: 9 GI, 27 GII, 3 GIII, 2 GIV, 2 GV, 2 GVI and 1 genotype each for GVII, GVIII, GIX (formerly GII.15) and GX [1]. Genogroups GI, GII and GIV are known to infect humans, with GII being the most common enteric pathogen responsible for acute gastroenteritis outbreaks worldwide and is frequently detected in environmental water sources [2]. Indeed, noroviruses are frequently identified in a diverse range of water sources, including groundwater, surface water, drinking water, tap water, rivers, swimming pools and wastewater [3]. According to the U.S. CDC, noroviruses were among the top five agents responsible for outbreaks linked to drinking water, both public and private, in the United States during the 2010–2022 period (https://www.cdc.gov/drinking-water/data-research/facts-stats/index.html) (accessed on 6 August 2024). This underscores the critical importance of monitoring water systems to mitigate the risks of waterborne outbreaks. Noroviruses are also the leading cause of foodborne outbreaks worldwide, responsible for a significant burden of gastroenteritis associated with contaminated food products [4]. These viruses are highly resilient and persist on food surfaces and in processing facilities, posing a critical challenge to food safety and public health authorities [5]. Outbreaks often stem from shellfish (especially bivalve mollusks), fresh produce (e.g., leafy greens, berries) and ready-to-eat foods prepared by infected handlers [6]. Their low infectious dose and rapid spread potential can lead to large-scale outbreaks in settings such as restaurants and cruise ships (https://www.cdc.gov/norovirus/outbreak-basics/?CDC_AAref_Val=https://www.cdc.gov/norovirus/outbreaks/common-settings.html) (accessed on 2 January 2025). Recent outbreaks have highlighted the importance of hand hygiene, environmental cleaning and exclusion of symptomatic food handlers as key prevention strategies [5,7]. Given the significant public health impact of HNoVs, effective surveillance is essential to monitor their circulation, identify emerging strains and mitigate outbreaks. In Europe, noroviruses are monitored through voluntary-based networks such as the “NoroNet”, a platform established in 1999 that promotes collaborative surveillance and research on enteric virus infections, with a primary focus on norovirus (https://www.rivm.nl/en/noronet) (accessed on 17 September 2019). In the United States, a similar effort is coordinated by the CDC through the “Calicinet”, a surveillance network dedicated to monitoring norovirus outbreaks and supporting public health interventions (https://www.cdc.gov/norovirus/php/reporting/calicinet.html) (accessed on 3 June 2024). Despite their significant impact on public health, there remains a lack of systematic characterization of HNoV gastroenteritis cases, particularly at the genotypic level. Clinical surveillance is often limited by underreporting, as not all cases of gastroenteritis result in medical consultations, and diagnostic testing is not always performed or accessible. As a result, clinical data alone provide an incomplete picture of HNoV circulation, leaving gaps in our understanding of the diversity and prevalence of genotypes responsible for infections. Environmental surveillance, particularly through the analysis of urban wastewater, provides a complementary and powerful tool to address these gaps. Wastewater collects pathogens, metabolites and AMR markers shed by the population it serves, providing a non-invasive and cost-effective method to monitor HNoV circulation within a community. Unlike clinical surveillance, wastewater monitoring can detect asymptomatic and subclinical infections, providing a more comprehensive overview of genotypic diversity and trends. By characterizing HNoV genotypes in wastewater, we can gain critical insight into their temporal dynamics, identify emerging strains and better understand their role in foodborne and waterborne outbreaks. Wastewater-based epidemiology (WBE), recognized as a valuable tool in tracking viral pathogens since the late 1990s, was first applied to HNoV in 1999, marking a pivotal step in understanding its circulation [8]. Since then, numerous studies worldwide have demonstrated the utility of this approach for investigating the genetic diversity of HNoV and observing population trends [9,10]. In Italy, no systematic clinical surveillance exists for norovirus-related gastroenteritis. The available information primarily comes from outbreak reports submitted to EFSA and efforts by the Italian Study Group for Enteric Viruses (ISGEV). ISGEV primarily focuses on research and sporadic studies rather than consistent and systematic monitoring, leaving significant gaps in understanding HNoV circulation within the population. Therefore, these data are fragmented and insufficient to provide a comprehensive epidemiological picture. In this context, wastewater-based epidemiology (WBE) emerges as a crucial approach to gathering genotypic information and assessing HNoV circulation in the population. Despite its potential, few studies have been conducted in Italy, and these have been limited to specific regions and timeframes [11,12,13,14]. This study aims to address some of these gaps. Building on our previous research conducted between 2011 and 2016 [14], we extend surveillance to cover the period from 2017 to 2024, analyzing HNoV circulation in wastewater in Rome. This prolonged timeframe provides a unique opportunity to track long-term trends, investigate the persistence and evolution of significant genotypes such as GII.4 and GII.17 and identify emerging variants. A key innovation of our study is the inclusion of the COVID-19 pandemic period, which introduced significant epidemiological and societal shifts. This allowed us to explore how such changes may have influenced HNoV circulation. The integration of nearly two decades of data facilitates comparative analyses, highlighting shifts in genotype prevalence and diversity over time and offering unprecedented insights into the long-term epidemiological dynamics of HNoVs in an urban setting. Methodologically, we employed next-generation sequencing (NGS) to investigate HNoV prevalence and diversity. NGS enables detailed identification of viral genotypes and genetic variations, offering significant advantages over traditional molecular methods. It facilitates the detection of mixed infections, rare genotypes and novel variants. Using this approach, our study aims to provide a deeper understanding of HNoV genotypic diversity and its circulation dynamics within the population.

## 2. Materials and Methods

### 2.1. Wastewater Sampling

From 2017 to 2024, a total of 337 wastewater 24 h composite samples were collected from five wastewater treatment plants (WWTPs) in Rome, chosen to represent different catchment areas of the city: 2017 (*n* = 28), 2018 (*n* = 52), 2019 (*n* = 33), 2020 (*n* = 36), 2021 (*n* = 50), 2022 (*n* = 37), 2023 (53), 2024 (*n* = 48). The variation in the number of samples collected each year reflects several factors, including logistical constraints and operational priorities. Figure S1 shows a geographic information system map of the WWTPs included in the study.

### 2.2. Viral Concentration and Nucleic Acid Extraction

Over the extended study period (2017–2024), which included the COVID-19 pandemic, the methodology for viral concentration underwent necessary modifications and improvements. From 2017 to 2019, samples were subjected to a two-phase separation method (polyethylene glycol (PEG)—dextran) in accordance with the World Health Organization (WHO) guidelines for poliovirus environmental surveillance, as [15] published [16]. Viral nucleic acid extraction was conducted on 5 mL of chloroform-treated samples using the NucliSENS MiniMag (bioMerieux, Marcy l’Etoile, France) semi-automated extraction system in accordance with the manufacturer’s instructions. The eluted RNA (100 μL) was stored in aliquots at −80 °C until molecular analysis was conducted.

From 2020 onward, a standardized national protocol was adopted, using a polyethylene glycol (PEG)-based method for viral concentration because it offered a more rapid and user-friendly alternative to the previously employed two-phase separation method [17]. Briefly, following an inactivation treatment at 56 °C for 30 min, 45 milliliters of the samples were concentrated using a PEG-based method [18]. Centrifugation at 4500× *g* for 30 min was employed to remove larger particles and debris. Subsequently, the supernatant (40 mL) was mixed with 8% polyethylene glycol 8000 and 0.3 M NaCl. Subsequently, the mixture was subjected to centrifugation at 12,000× *g* for a period of 2 h. After centrifugation, the supernatant was discarded, and the pellet was resuspended in 2 mL of PBS for extraction. The extraction of nucleic acids was conducted using magnetic silica-based systems that were fully automated, and the samples were subsequently purified with the OneStep PCR Inhibitor Removal Kit (Zymo Research USA, Irvine, CA, USA). Purified RNAs were then stored at −80 °C.

### 2.3. Next-Generation Sequencing of Genogroup GI and GII

Following RNA extraction, all samples underwent PCR amplification by nested RT-PCR for HNoV genogroup I and II. The nested RT-PCR employed primer sets, previously described [19,20], are designed to amplify conserved region C (capsid gene) of the HNoV genome. The amplicon size was 330 bp for GI and 344 bp for GII, respectively. Detailed primer sequences and thermocycling conditions are provided in Supplementary Table S1. First-strand cDNA was synthesized using Super Script IV Reverse Transcriptase (Thermo Fisher Scientific, Waltham, MA, USA) using the reverse primer specific for HNoV genogroups I and II, according to the manufacturer’s instructions. The PCR reaction was performed using 2 μL of synthesized cDNA in a final volume of 25 μL. The reaction mixture included Platinum SuperFi Green PCR Master Mix (Thermo Fisher Scientific) and 1 μL of each primer (10 μM). After the first round of PCR, a nested PCR was performed using 1 μL of the first PCR product as the template. The nested PCR was carried out under the same reaction conditions as the first PCR. Positive controls for both genogroups (GI and GII) were included in each assay, using reference strains of HNoV GI.4 and GII.4, respectively, derived from a collection of stool samples from pediatric patients with gastroenteritis. Negative controls were also included in each assay to monitor potential contamination and ensure assay reliability. PCR products were visualized by gel electrophoresis, and those yielding the correct amplicon size were purified using Montage PCRm96 Micro well Filter Plates (Millipore, Burlington, MA, USA) to remove primers, enzymes and other reaction components before downstream applications. The PCR amplicons generated using broad-range primers were combined into 8 pools: one for each year of sampling containing both GI and GII products. Equal amounts of purified PCR amplicons from each sample were mixed to create each pool. The pooled DNA was then precipitated with isopropanol and the pellet was resuspended in nuclease-free water to a final volume of 20 µL. The DNA concentration and purity of each pool were assessed by measuring A260/A280 ratios using a Nanodrop 1000 spectrophotometer and Qubit RNA HS Assay (Thermo Fisher Scientific). These steps ensured that the DNA was of sufficient quality and quantity for subsequent NGS analysis. NGS was carried out on a MiSeq II sequencer (Illumina, San Diego, CA, USA) according to the manufacturer’s instructions. For the library preparation, 5 ng of PCR amplicons were simultaneously fragmented and tagged with sequencing adapters (Nextera XT DNA Library Preparation Kit and Nextera XT Index Kit). The following run of paired-end sequencing, cluster generation and sequencing by synthesis was performed using the MiSeq Reagent Kit v3 (600-cycle, extend read lengths up to 2 × 300 bp). Base calls generated on-instrument were first processed by Real-Time Analysis (RTA v1.18.54, Illumina) software to assess quality scores and then passed through the MiSeq Reporter (MSR) software (v4.1.0) to generate the initial rough alignment, structural variants and contig assemblies for each sample (Resequencing pipeline). A no-template control (NTC) was included in the Nextera XT Library Procedures to verify the absence of contaminant amplifications. A V3 PhiX library was also included in each MiSeq run for sequencing quality control.

### 2.4. Bioinformatic Analysis

A similarity-based approach was employed for genotype and for GII.4 variant identification. For this purpose, reference sequences from the Human Calicivirus Typing Tool were used, available at https://calicivirustypingtool.cdc.gov/becerance.cgi (accessed on 14 November 2024), as shown in Tables S2 and S3. In total, 55 sequences were used for HNoV GI and 136 for HNoV GII. To guide the selection of an appropriate similarity threshold, an all-vs.-all BLAST (Basic Local Alignment Search Tool) comparison was performed within the reference genotypes [21] (Figures S2 and S3). This analysis assessed similarity percentages among genotypes of genogroups I and II, respectively, enabling the determination of a similarity threshold that reliably discriminated genotypes. Using this approach, the thresholds were evaluated and summarized in Table S4, ensuring a balance between inclusivity (correct identification of target variants) and exclusivity (accurate differentiation from non-target sequences). Subsequently, paired-end reads, once merged, were directly aligned to the reference database to determine the virus genotype. The tool Ampliconseq v1.0.0 (GNU GPLv3) [22], executed under Conda 4.9.2, was used to implement the desired analysis pipeline. The tool was run with default parameters, producing joined reads with an average Phred quality score of 37, indicating very high sequence quality. Only full-length reads that matched the length of the amplicon reference sequence were considered positive. For the assignment of GII.4 variants, the Norovirus Typing Tool from RIVM, available at https://www.rivm.nl/mpf/typingtool/norovirus/ (accessed on 17 September 2019), was used. Specifically, 100 reads randomly assigned to GII.4 from each year were selected and organized into a FASTA file, which was then uploaded to the website for analysis.

## 3. Results

### 3.1. Positivity for GI and GII over the Study Period

During the study period, amplification of 330-bp and 344-bp fragments for HNoV-GI and HNoV-GII was obtained in 171/337 (50.7%) and 224/337 (66.5%) samples, respectively, using RT-nested PCR (Table 1 and Table S5). All negative and positive controls produced the expected results. Table 1 shows the number of positive samples and percentages (%) detected by RT-nested PCR per year. Over the study period, the percentages of positive samples for HNoV-GI and HNoV-GII varied significantly across years. HNoV-GII consistently exhibited higher detection rates than HNoV-GI in most years. The lowest detection rate for HNoV-GI (17.8%) was observed in the first year of the study, whereas HNoV-GII had a relatively high positivity rate (64.3%). In 2019, HNoV-GII detection reached its highest level (90.9%), while HNoV-GI positivity also peaked at 60.6%.

In 2020, a significant decline in positivity was observed for both genogroups, with HNoV-GI dropping to 25.0% and HNoV-GII to 19.4%. This drop coincided with the COVID-19 pandemic. Finally, in 2023, HNoV-GI exhibited its highest detection rate (77.3%), while HNoV-GII also had a high positivity rate (75.5%).

### 3.2. Results of Sequencing and Genotype Characterization

The sequencing of the eight samples representing the eight-year pools generated an average of 404,589 paired-end raw reads. Using the Ampliconseq v. 1.0.0 tool, these raw reads were processed, yielding a mean of 235,136 joined reads per sample. The resulting reads were subsequently used for sequence similarity assignment. The all-vs.-all BLAST analysis revealed that a genetic identity cut-off of 87.5% was sufficient for accurate discrimination of all genotypes within genogroup GI (Figure S2). However, minor issues were observed for GI.3 and GI.5, where this threshold resulted in a potential (though negligible) loss of assigned reads based on the reference sequences used as prototype strains. For genogroup GII, discrimination of genotypes required a tailored approach. Three identity cut-off thresholds—87.5%, 90.5% and 92.5%—were applied depending on the specific genotypes (Figure S3). Similarly to genogroup GI, potential loss of assigned reads was observed for certain genotypes, such as GII.6 and GII.17. Nevertheless, specificity was consistently maintained, ensuring accurate genotype discrimination even in cases where minor read losses occurred. Figures S2 and S3 in the Supplementary Materials provide detailed insights into the BLAST results and the threshold selection process for GI and GII, respectively. The selected percentages for each genotype are shown in Table S4.

**Figure 1:**
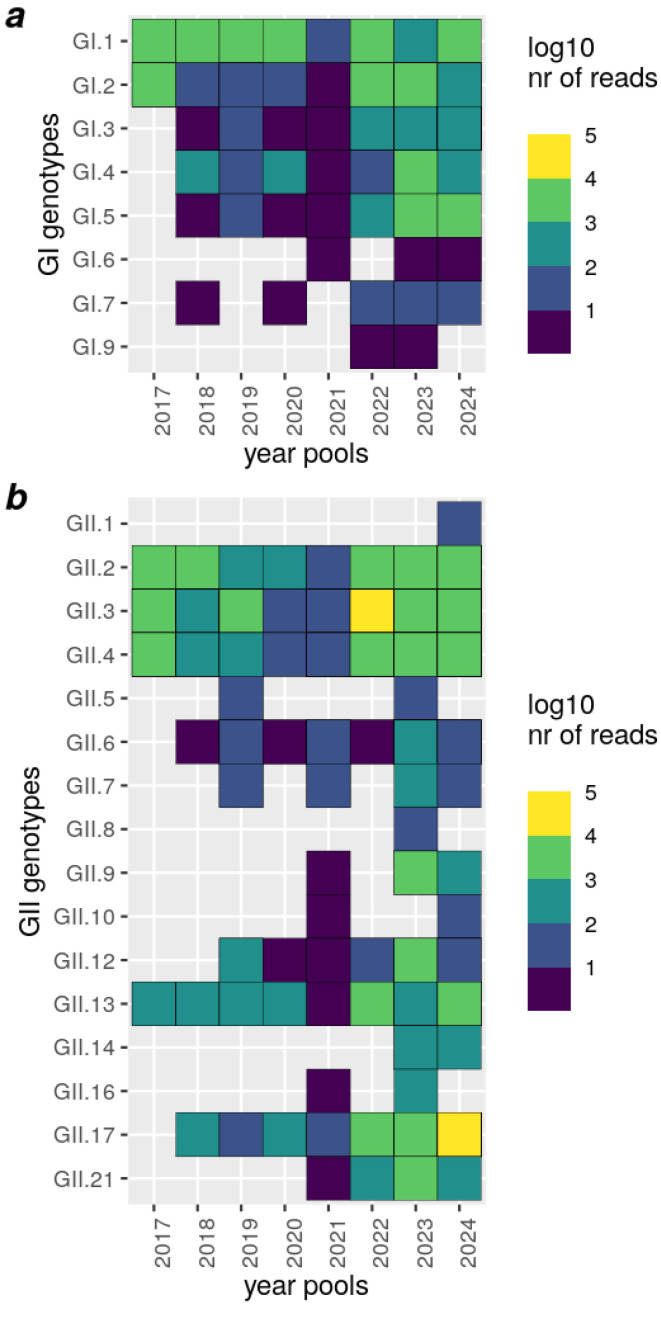
Heatmap representing relative abundance and temporal dynamics of genogroups GI (**a**) and GII (**b**) and their respective genotypes across the eight-year study period. X-axis: year pools from 2017 to 2024. Y-axis: list of genotypes.

Table 2 summarizes the annual distribution of genogroups GI and GII and their genotypes across the study period. For genogroup I (GI), eight genotypes were identified, namely GI.1, GI.2, GI.3, GI.4, GI.5, GI.6, GI.7 and GI.9. Among these, GI.1 and GI.2 were consistently detected across all years, highlighting their stable circulation. Over the 8-year period, the GI genogroup showed a gradual increase in diversity, starting from two genotypes in 2017 to a consistent presence of eight genotypes by 2023–2024. Genotypes GI.3, GI.4 and GI.5 appeared from 2018 onward, showing stable detection, while GI.6, GI.7 and GI.9 were more sporadic, with their first appearances occurring later in the timeline (2021 for GI.6, 2018 for GI.7 and 2022 for GI.9). About genogroup GII, GII.2 and GII.3 were consistently detected every year from 2017 to 2024, highlighting their persistent circulation and widespread presence. In contrast, other genotypes appeared intermittently or emerged later in the timeline. Genotypes such as GII.5, GII.7, GII.9, GII.10, GII.12, GII.13 and GII.17 were detected across multiple years, while GII.8, GII.14, GII.16 and GII.21 emerged only in the later years of the study, particularly between 2022 and 2024. The period from 2023 to 2024 represented the peak in genotype diversity, with up to 13–14 distinct GII genotypes identified, including newly emerging entries such as GII.8 and GII.14. Regarding the GII.4 variants of the 718 sequences in the FASTA file, the Norovirus Typing Tool was unable to assign 122 sequences. The remaining sequences were classified as either GII.4 Sydney_2012 (*n* = 75, closest accession numbers: JX459908 and JX459907) or GII.4 Sydney_2016 (*n* = 521, closest accession numbers: LC153121 and LC165468) (Table 2; Figure 1).

**Table 2:** Summary of the genotypes detected in each year.

Genogroup	Genotype	Year							
		2017	2018	2019	2020	2021	2022	2023	2024
GI	GI.1	×	×	×	×	×	×	×	×
	GI.2	×	×	×	×	×	×	×	×
	GI.3		×	×	×	×	×	×	×
	GI.4		×	×	×	×	×	×	×
	GI.5		×	×	×	×	×	×	×
	GI.6					×		×	×
	GI.7		×		×		×	×	×
	GI.9						×	×	
	Total Genotypes	2	6	5	6	6	7	8	7
GII	GII.1								×
	GII.2	×	×	×	×	×	×	×	×
	GII.3	×	×	×	×	×	×	×	×
	GII.4	×	×	×	×	×	×	×	×
	GII.5			×					
	GII.6		×		×	×	×	×	×
	GII.7			×		×		×	×
	GII.8							×	
	GII.9					×		×	×
	GII.10					×			×
	GII.12			×	×	×	×	×	×
	GII.13	×	×	×	×	×	×	×	×
	GII.14							×	
	GII.16					×		×	
	GII.17		×	×	×	×	×	×	×
	GII.21					×	×	×	×
	Total Genotypes	4	6	8	7	12	8	13	12

The heatmap in Figure 1a illustrates the relative abundance of GI genotypes over the eight years of the study period. The X-axis represents the pooled years, while the Y-axis lists the detected GI genotypes. GI.2 and GI.3 were consistently detected across multiple years, specifically in 2018, 2020, 2021 and 2023, showing high read counts, as indicated by the yellow and green squares. GI.4 and GI.5 appeared intermittently, with notable read abundance in 2021 and 2022. In contrast, GI.6, GI.7 and GI.9 were less frequently detected and exhibited lower read counts, as represented by the predominance of blue and purple shades. The heatmap in Figure 1b depicts the relative abundance of GII genotypes over the eight-year study period. Compared to GI, the GII genogroup exhibited greater complexity, both in terms of the number of genotypes and their distribution over time. Specifically, GII.2, GII.3, GII.4 and GII.17 stood out as the most abundant genotypes (yellow squares), with high read counts in specific years, particularly during 2021 and 2023. Other genotypes, including GII.3 and GII.6, appeared intermittently but displayed moderate to high read counts in specific years. In contrast, certain genotypes, such as GII.1, GII.8, GII.9, GII.10 and GII.14, showed sporadic detection with generally lower read abundances, as indicated by the blue and purple shades. The GII.4 subvariants were particularly notable for their persistent presence over multiple years. According to the Norovirus Typing Tool (RIVM), the Sydney 2012 and Sydney 2016 strains predominated throughout the eight years of surveillance. 

## 4. Discussion

Human Norovirus is a leading cause of acute gastroenteritis worldwide, causing a significant burden of illness across all age groups. Its infectivity and capacity to cause outbreaks in a range of settings, from healthcare facilities to cruise ships, represents a significant public health challenge. The rapid transmission of HNoV, coupled with its significant morbidity, particularly in vulnerable populations such as young children, the elderly and immunocompromized individuals, highlights the need to monitor its spread and genetic evolution. Furthermore, the virus’s remarkable genetic diversity, driven by frequent recombination events between polymerase and capsid regions [23], presents additional challenges for vaccine development and outbreak management. Additionally, the ability of HNoV to persist in the environment and its extremely low infectious dose further emphasize the importance of monitoring this pathogen as a focus for public health surveillance [24].

Wastewater serves as a crucial reservoir for HNoV, capturing human shedding patterns and providing population-level infection trends. This study, conducted from 2017 to 2024, analyzed wastewater samples from sewage treatment plants in Rome over an eight-year period to evaluate the genetic diversity of HNoV genogroups GI and GII, with a focus also on the GII.4 variants circulating in the study area during this timeframe. Building on previous surveillance efforts [14], this study extended HNoV monitoring in wastewater, providing insights into long-term trends. 

Our findings revealed that HNoV detection rates fluctuated considerably across the study period, influenced by both intrinsic viral dynamics and external factors. Genogroup GII consistently exhibited higher positivity rates (66.5% on average over the study period) compared to GI (50.7%). Our findings are in line with other studies on HNoV epidemiology. The predominance of GII genotypes over GI is a well-documented global trend and has been consistently reported in various studies. This pattern reflects the greater epidemiological impact and evolutionary success of GII strains, particularly in causing large-scale outbreaks [2]. The detection rates peaked in 2019 for both genogroups (HNoV-GI: 60.6%; HNoV-GII: 90.9%) before experiencing a significant drop in 2020, coinciding with the COVID-19 pandemic. This decline is likely attributable to non-pharmaceutical interventions such as lockdowns, social distancing and enhanced hygiene measures, which disrupted HNoV transmission pathways [25,26]. Notably, as restrictions eased post-pandemic, HNoV circulation rebounded, with HNoV-GI reaching its highest detection rate in 2023 (77.3%) and HNoV-GII maintaining elevated levels (75.5%). These findings emphasize the responsiveness of wastewater surveillance in capturing shifts in viral transmission during periods of societal change. Similar results have been documented in England, where increases in HNoV levels in wastewater appeared to coincide with the removal of COVID-19-related lockdown restrictions [27]. Our findings revealed a notable prevalence of diverse HNoV genotypes from both genogroups in raw sewage. Over the eight-year monitoring period, a total of 25 genotypes were identified, comprising 8 genotypes within GI and 15 within GII, highlighting the extensive genetic diversity of circulating HNoVs in Rome. For genogroup GI, genotypes GI.1 and GI.2 were consistently detected throughout the study, suggesting stable circulation within the population. In contrast, other genotypes, such as GI.3, GI.4 and GI.5, emerged later and exhibited sporadic detection patterns. The gradual increase in GI diversity, particularly during the post-pandemic period (2022–2024), may reflect shifts in viral dynamics.

In genogroup GII, the predominance of genotypes GII.2, GII.3, GII.4 and GII.17 was particularly salient. These genotypes are recognized for their high transmissibility and association with global outbreaks, underscoring their epidemiological significance [28]. On a global scale, genotype GII.4 predominates, with new variants emerging at a rate of 2 to 5 years [29]. Since the 1990s, six major epidemic GII.4 variants have emerged: Grimsby 1995, Farmington Hills 2002, Hunter 2004, Den Haag 2006, New Orleans 2009 and Sydney 2012. These variants have been observed to occur in a chronological sequence, a phenomenon that may be attributed to the influence of selective pressures from herd immunity against preceding variants [30]. As for the GII-4 variants, only GII.4 Sydney_2012 and GII.4 Sydney_2016 were detected in this study. Since 2012, the GII.4 Sydney 2012 variant has been the most frequently and predominantly detected strain in wastewater samples [9]. Molecular epidemiological analyses of HNoVs have confirmed that GII.4 Sydney 2012 remains the primary strain responsible for acute gastroenteritis (AGE) outbreaks across most regions worldwide [31,32,33]. This is also the case in Italy, where from 2011 to 2020, GII.4 Sydney 2012 circulated stably [34]. 

Another interesting genotype is GII.17. It has circulated since 1978 [35] but has been successively sporadically detected in Europe, Africa, America, Australia and Asia. During the 2014–2015 season, the GII.17_2014 (Kawasaki) pandemic variant emerged, and it was responsible for gastroenteritis outbreaks in schools, colleges, factories and kindergartens in China, where 82.8% of the outbreaks reported were caused by GII.17_2014. This variant has been associated with outbreaks of gastroenteritis in several countries and, in some cases, replaced the dominant GII.4 Sydney 2012 genotype [36]. In Italy, the GII.17 variant was first reported in a case of acute severe gastroenteritis (AGE) in young children [37] and subsequently in other pediatric cases of acute severe gastroenteritis [38], suggesting an increase in circulation. However, GII.17 Kawasaki 2014 has been circulating in the population since 2013 before being first reported by wastewater monitoring [14,39], highlighting the importance of this approach. This variant was also identified in shellfish samples collected in 2015, providing indirect evidence that this strain was present in the Italian population prior to the first detection of clinical cases [40]. These findings are consistent with global reports describing GII’s capacity to generate novel variants and drive localized outbreaks [23].

In Italy, there is limited information available regarding the circulation of HNoVs and their genotyping. For instance, data from surveillance in an AGE study conducted in Palermo on gastroenteritis cases from 1986 to 2020 (partially overlapping with the period of our monitoring) of hospitalized viral symptomatic children revealed that the most prevalent genotype was GII.4 (60.8%) followed by GII.3 (13.3%), GII.2 (12.4%), GII.6 (4.7%), GII.17 (2%) and GII.1 (1.9%) [34]. In relation to environmental surveillance, the 14-year monitoring period encompasses data from two studies: the initial study, which was conducted between 2011 and 2016 and has been previously published [14], and the ongoing study, which extended from 2017 to 2024. Specifically, for genogroup GII, the data from these two studies revealed the consistent presence of several genotypes over time. Genotypes such as GII.1, GII.2, GII.3, GII.4, GII.6, GII.7, GII.13, GII.17 and GII.21 were detected throughout both studies, indicating their persistent circulation across the entire 14-year period. Conversely, new genotypes, including GII.8, GII.9, GII.10, GII.12 and GII.14, were exclusively identified during the 2017–2024 monitoring period, suggesting an expansion in the diversity of circulating HNoV strains in more recent years. These findings underscore the stability of certain genotypes and the emergence of new ones, emphasizing the evolving genetic variability of HNoV. 

While this study provides valuable insights into HNoV circulation in Rome, certain limitations must be acknowledged. First, the variability in sampling frequencies across years may have influenced the observed trends. Second, the reliance on pooled sequencing could obscure low-abundance genotypes. Additionally, expanding WBE efforts to other regions of Italy and integrating clinical data would provide a more comprehensive understanding of HNoV epidemiology nationwide. Given the ongoing evolution of HNoVs and their impact on public health, sustained surveillance is essential to identify emerging variants, assess their epidemiological significance and support the development of effective vaccines and control measures. Another limitation of this study is that we sequenced only the capsid-coding region, which prevents us from detecting potential recombinant strains. Additionally, the pooling of annual samples hindered the ability to analyze seasonality, as the temporal resolution of seasonal trends was lost. 

A primary advantage of our study is its extensive eight-year monitoring period, a notable distinction from most studies that restrict their sampling to approximately one year [9]. The extended observation period enables a more comprehensive examination of trends in individual HNoV genotypes, facilitating a more profound comprehension of local characteristics and the distribution of dominant genotypes. This, in turn, provides invaluable insights into the long-term epidemiological dynamics of HNoV. A particularly notable finding is our observation of genogroup GI, which was expanded. In the 2011–2016 monitoring period [14], the GII genogroup was the primary focus. 

## 5. Conclusions

The findings of this study demonstrated that the sequencing of HNoV genotypes in urban wastewater serves as an effective approach for monitoring the circulation of viral strains within the population.

## Figures and Tables

**Table 1:** Prevalence of HNoV-GI and HNoV-GII positive samples amplified by RT-nested PCR for each year.

Sampling Year	HNoV-GI		HNoV-GII	
	Positive/Total	Percentage (95% C.I.)	Positive/Total	Percentage (95% C.I.)
2017	5/28	17.8% (3.6–32.0%)	18/28	64.3% (46.5–82.0%)
2018	28/52	53.8% (40.3–67.3%)	38/52	73.0% (61.0–85.1%)
2019	20/33	60.6% (43.9–77.3%)	30/33	90.9% (81.1–100.7%)
2020	9/36	25.0% (10.9–39.1%)	7/36	19.4% (6.5–32.4%)
2021	11/50	22.0% (10.5–33.5%)	39/50	78.0% (66.5–89.5%)
2022	26/37	70.3% (55.5–85.0%)	17/37	45.9% (29.9–62.0%)
2023	41/53	77.3% (66.1–88.6%)	40/53	75.5% (63.9–87.1%)
2024	31/48	(64.6%) (51.1–78.1%)	35/48	72.9% (60.3–85.5%)
Total	171/337	59.7% (54.4–64.9%)	224/337	66.5% (61.4–71.5%)

## Data Availability

The original contributions presented in this study are included in the article/Supplementary Materials. Further inquiries can be directed to the corresponding authors.

## Supplementary Materials

The following supporting information can be downloaded at: https://www.mdpi.com/article/10.3390/v17010130/s1, Figure S1. Geographic information system map of the WWTPs included in the study; Figure S2. BLAST results and the threshold selection process for genogroup GI. The selected percentages for each genotype are shown; Figure S3. BLAST results and the threshold selection process for genogroup GII. The selected percentages for each genotype are shown; Table S1. PCR primers and amplified products used in this study; Table S2. Reference sequences (N = 55) from the Human Calicivirus Typing Tool (https://calicivirustypingtool.cdc.gov/becerance.cgi, accessed on 14 November 2024) for genotype GI; Table S3. Reference sequences (N = 136) from the Human Calicivirus Typing Tool (https://calicivirustypingtool.cdc.gov/becerance.cgi, accessed on 14 November 2024) for genotype GII; Table S4. Selected cut-off threshold (%) for each genotype; Table S5. Results obtained from RT-nested PCR for NoV-GI (330-bp fragment) and NoV-GII (344-bp fragment).

## Abbreviations

The following abbreviations are used in this manuscript:

HNoV	Human Norovirus
WBE	Wastewater Based Epidemiology
WWTPs	Wastewater Treatment Plants
GI	Genogroup I
