# Supplementary material for: Eight Years of Norovirus Surveillance in Urban Wastewater: Insights from Next-Generation
Source: Viruses. 2025 Jan 17;17(1):130. doi: 10.3390/v17010130 (PMC11768713; doi:10.3390/v17010130)
Supplement: Supplementary file 1 [file viruses-17-00130-s001.zip › viruses-3422435-Supplementary Materials.pdf]

Supplementary material

Figure S1. Geographic information system map of the WWTPs included in the study.

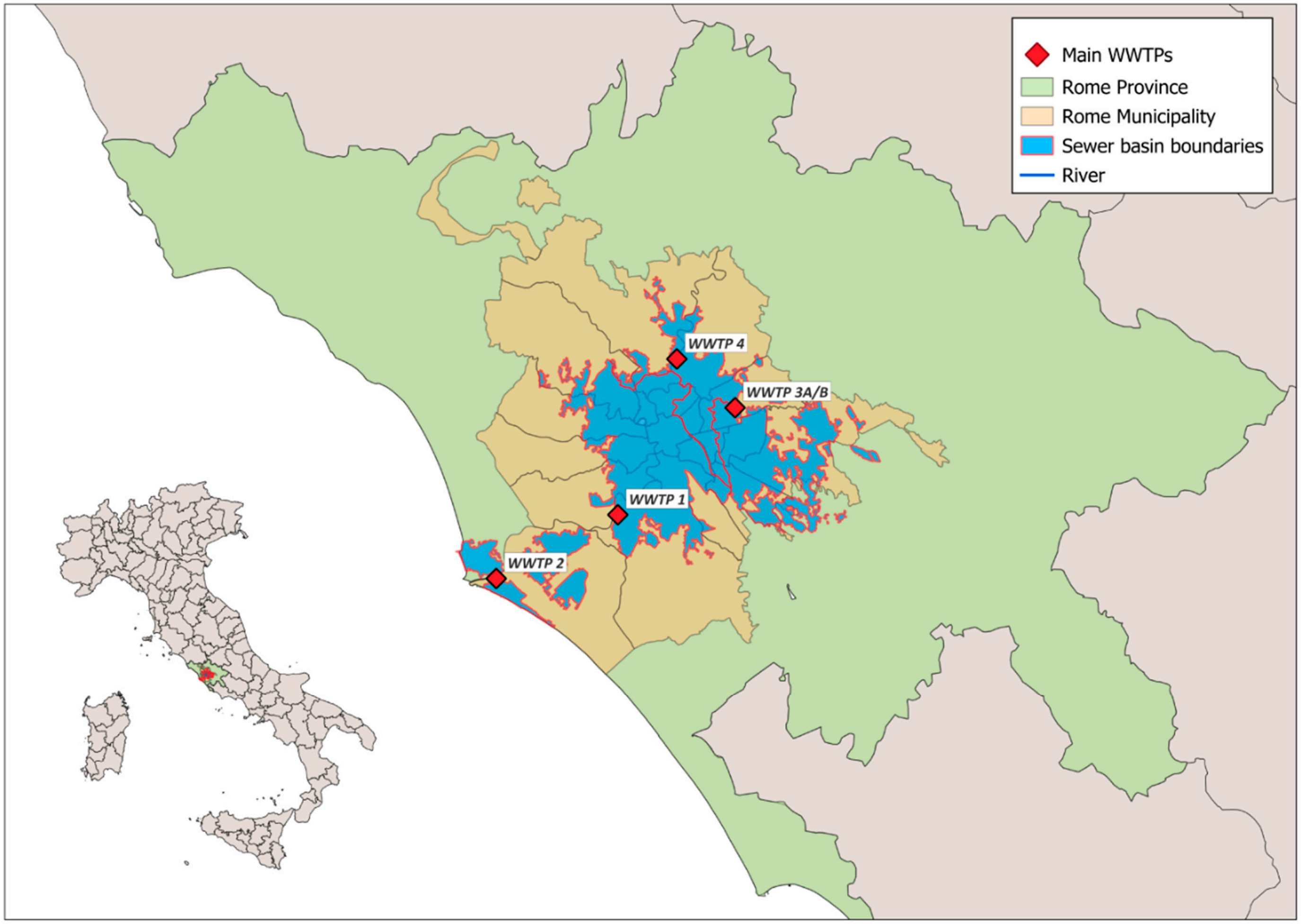

Table S1. PCR primers and amplified products used in this study.

Virus	Target region	Primer name	Primer sequence (5′ - 3′)	Lengh (bp)	PCR	Annealing Temperature (C°)	Refrences
**NoV GI**	ORF2	COG1F	CGY TGG ATG CGN TTY CAT GA	381	1st cycle	50*	[Kageyama et al., 2003; Kojima et al., 2002]
		G1-SKR	CCA ACC CAR CCA TTR TAC ATT T				
		G1-SKF	CTG CCC GAA TTY GTA AAT GA	330	nested-PCR	50 *	
		G1-SKR	CCA ACC CAR CCA TTR TAC ATT T				
**NoV GII**	ORF2	COG2F	CAR GAR BCN ATG TTY AGR TGG ATG AG	387	1st cycle	52 *	[Kageyama et al.,2003; Kojima et al., 2002]
		G2-SKR	CCR CCN GCA TRH CCR TTR TAC AT				
		G2-SKF	CNT GGG AGG GCG ATC GCA A	344	nested PCR	50 *	
		G2-SKR	CCR CCN GCA TRH CCR TTR TAC AT				

*The PCR conditions were as follows: 98 °C for 30 s; 35 cycles at 98 °C for 10 s, 50/52 °C for 10 s, and 72 °C for 30 s; final extension at 72 °C for 5 min

Table S2. Reference sequences (N=55) from the Human Calicivirus Typing Tool (https://calicivirustypingtool.cdc.gov/becerance.cgi) for genotype GI

Norwalk	M87661	G1.1
Gothenburg	EU085529	
SRSV-KY-8989J	L23828	
V1622-06	AB447406	
Southampton	L07418	GI.2
Constellation59	AF435807	

Leuven	FJ515294	
DesertShield395	U04469	GI.3
Akabane991130	EF547396	
Beijing54108	GQ856470	
Beijing54114	GQ856471	
Beijing54660	GQ856472	
Beijing55042	GQ856473	
CHDC116	JX846929	
Muenster702	KM289169	
Otofuke	AB187514	
Potsdam196	AF439267	
ShimizuKK2866	KJ196292	
Stavanger	AF145709	
V170707	AB447414	
VA98115	AY038598	
Vesoul576	EF529738	
Chiba407	AB042808	GI.4
Beijing55169	GQ856475	
Groningen	LN854563	
Koblenz433	AF394960	
Queensarmsleeds92	AJ313030	
Musgrove	AJ277614	GI.5
AppalachicolaBay318	AF414406	
OC020180	LC101825	
Siklos-HUN5407	KJ402295	
SzUG1	AB039774	

Yiwu-CB1	MH443711	
BS5(Hesse)	AF093797	GI.6
14-BC-1	KP027330	
Beijing53997	GQ856463	
Beijing55063	GQ856464	
Hiroshima48-938	AB354289	
No20-Saitama-98-17	LC342057	
Sindlesham	AJ277615	
VA497	AF538678	
WUG1	AB081723	
Winchester	AJ277609	GI.7
AlbertaEI404	KU311161	
Chiba030100	AJ844469	
Dhaka1882	MH130046	
IF2036	AY675555	
LillaEdetS5b	JN603251	
NizhnyNovgorod	FJ383816	
Providence	JN899243	
Boxer	AF538679	GI.8
NagoyaKY531	KJ196298	
Vancouver730	HQ637267	GI.9
CAIQ12110628	KF586507	
LillaEdetS48	JN183159	

M87661 EU085529 L23828 AB447406 L07418 AF435807 FJ515294 U04469 EF547396 GQ856470 GQ856471 GQ856472 GQ856473 JX846929 KM289169 AB187514 AF439267 KJ196292 AF145709 AB447414 AY038598 EF529738 AB042808 GQ856475 LN854563 AF394960 AJ313030 AJ277614 AF414406 LC101825 KJ402295 AB039774 MH443711 AF093797 KP027330 GQ856463 GQ856464

AB354289 LC342057 AJ277615 AF538678 AB081723 AJ277609 KU311161 AJ844469 MH130046 AY675555 JN603251 FJ383816 JN899243 AF538679 KJ196298 HQ637267 KF586507 JN183159

Table S3. Reference sequences (N=136) from the Human Calicivirus Typing Tool (https://calicivirustypingtool.cdc.gov/becerance.cgi) for genotype GII

Hawaii	U07611	GII.1
Amsterdam3	KJ194507	
Ascension208	JN797508	
Dillingen391	AF425767	
Picton	AY919139	
Melksham	X81879	GII.2
E3	AY682552	
HenrytonSP17	MF405169	
KL109	JX846925	
MK04	DQ456824	
OC97007	AB089882	
OsakaNI	DQ366347	
PontdeRoide673	AY682549	
SaitamaE24	KF730316	
SantaRosa1764	KY865306	
SnowMountain	AY134748	
Vaals	AB281090	
Toronto	U02030	GII.3
109	MH218579	
Amsterdam1	KJ194500	
Arg320	AF190817	
HK71	JX846924	
Herzberg385	AF539439	
IPH2172-09VG4	JF697282	

MX	U22498	
Milwaukee009	JN565063	
NSW730O	KT239614	
RotterdamE1300327	MF140689	
RotterdamP1D0	AB385626	
SN2000JA	AB190457	
CHDC2094	FJ537135	GII.4
CHDC5191	FJ537134	
Asia	AB220921	GII.4 Asia
Sakai04179	AB220922	
DenHaag	EF126965	GII.4 Den Haag
Minerva	EU078417	
Shimane2	AB541348	
FarmingtonHills	AY502023	GII.4 Farmington Hills
Langen1061	AY485642	
CUHK-NS-2200	MN400355	GII.4 Hong Kong
Chiba04-1008	AB220926	GII.4 Hunter
Hunter504D04O	DQ078814	
Hunter	AY883096	
Hokkaido3	AB933767	GII.4 New Orleans
NSW001P	GQ845367	
NewOrleans_2010-SP-0870	KX353972	
NewOrleans	GU445325	
Osaka	AB434770	GII.4 Osaka
PunePC51	EU921388	
OH16002	LC153121	GII.4 Sydney

Sydney	JX459908	
WI-467121	KX354134	
WoononaNSW3309	JX459907	
Saitama	AB112306	GII.4 US95-96
US95-96	AF414424	
US95-96	AJ004864	
Isumi060936	AB294790	GII.4 Yerseke
Yerseke	EF126963	
Hillingdon	AJ277607	GII.5
S63	AY682550	
SaitamaT49	KJ196277	
SaitamaT52	KJ196288	
Seacroft	AJ277620	GII.6
14-55	KX158281	
30443	KM198534	
Belgium-4	MN248516	
GZ2010-L96Guangzhou	JX989075	
Miami292	AF414410	
S9c	KC576910	
SaitamaU16	AB039778	
SaitamaU4	AB039777	
Shizuoka8913	HM633213	
TCHE99-13646	GU930737	
Tokyo	AB684664	
Leeds	AJ277608	GII.7
236	MH218692	

CHDC3936	JX846926	
Gwynedd273	AF414409	
TAKAsanKimchi	KJ196295	
Amsterdam9818	AF195848	GII.8
SaitamaU25	AB039780	
VA97207	AY038599	GII.9
GoulburnValleyG5175C	DQ379715	
Erfurt546	AF427118	GII.10
Mc37	AY237415	
Sw918	AB074893	GII.11
Sw43	AB126320	
Wortley	AJ277618	GII.12
No35-Saitama-99-1	LC342059	
PunePC24	EU921353	
SaitamaU1	AB039775	
StGeorge	GQ845370	
Fayetteville	AY113106	GII.13
Empangeni10403	KR904229	
GoulburnValleyG5175B	DQ379714	
PunePC25	EU921354	
SaitamaT80	KJ196276	
M7	AY130761	GII.14
Beijing55028	GQ856465	
Maizuru8533	GU017903	
PV-Voelk0017	KM289171	
Saga8610	GU594162	

SendaiYG99	KJ196278	
Tiffin	AY502010	GII.16
Hiram	AY502006	
Neustrelitz260	AY772730	
CS-E1	AY502009	GII.17
Arg13099	KX061540	
Briancon870	EF529741	
CUHK-NS-682	KT589391	
Katrina-17	DQ438972	
Kawasaki308	LC037415	
Kawasaki323	AB983218	
SaitamaT87	KJ196286	
OH-QW101	AY823304	GII.18
OH-QW125	AY823305	
OH-QW170	AY823306	GII.19
Luckewalde591	EU373815	GII.20
Leverkusen267	EU424333	
OC071182007	AB542917	
IF1998	AY675554	GII.21
KawasakiYO284	KJ196284	
Yuri	AB083780	GII.22
Dhaka1940	MG495082	
Loreto1847	KT290889	GII.23
Loreto6422	MG495080	
Loreto1972	KY225989	GII.24
EdenPrairie5457	MG495084	

Beijing53931	GQ856469	GII.25
Dhaka1928	MG495083	
Leon4509	KU306738	GII.26
Leipzig07788a	MF352142	
Loreto0959	MG495077	GII.27
Arg15813	MK733205	
Loreto1257	MG495079	GII.NA1
PNV06929	MG706448	GII.NA2

U07611 KJ194507 JN797508 AF425767 AY919139 X81879 AY682552 MF405169 JX846925 DQ456824 AB089882 DQ366347 AY682549 KF730316 KY865306 AY134748 AB281090 U02030 MH218579 KJ194500 AF190817 JX846924 AF539439 JF697282 U22498 JN565063 KT239614 MF140689 AB385626 AB190457 FJ537135 FJ537134 AB220921 AB220922 EF126965 EU078417 AB541348 AY502023 AY485642 MN400355 AB220926 DQ078814 AY883096 AB933767 GQ845367 KX353972 GU445325 AB434770 EU921388 LC153121 JX459908 KX354134 JX459907 AB112306 AF414424 AJ004864 AB294790 EF126963 AJ277607 AY682550 KJ196277 KJ196288 AJ277620 KX158281 KM198534 MN248516 JX989075 AF414410 KC576910 AB039778 AB039777 HM633213 GU930737 AB684664 AJ277608 MH218692 JX846926 AF414409 KJ196295 AF195848 AB039780 AY038599 DQ379715 AF427118 AY237415 AB074893 AB126320 AJ277618 LC342059 EU921353 AB039775 GQ845370 AY113106 KR904229 DQ379714 EU921354 KJ196276 AY130761 GQ856465 GU017903 KM289171 GU594162 KJ196278 AY502010 AY502006 AY772730 AY502009 KX061540 EF529741 KT589391 DQ438972 LC037415 AB983218 KJ196286 AY823304 AY823305 AY823306 EU373815 EU424333 AB542917 AY675554 KJ196284 AB083780 MG495082 KT290889 MG495080 KY225989 MG495084 GQ856469 MG495083 KU306738 MF352142 MG495077 MK733205 MG495079 MG706448

Table S4. Selected cut-off threshold (%) for each genotype.

Genogroup	Genotypes	Cut-off Thresholds (%)
**GI**	G1.1	87.5%
	GI.2	
	GI.3	
	GI.4	
	GI.5	
	GI.6	
	GI.7	
	GI.9	
**GII**	GII.1	90.5%
	GII.2	87.5%
	GII.3	87.5%
	GII.4	87.5%
	GII.4 Asia	
	GII.4 Den Haag	
	GII.4 Farmington Hills	
	GII.4 Hong Kong	
	GII.4 Hunter	
	GII.4 New Orleans	
	GII.4 Osaka	
	GII.4 Sydney	

	GII.4 US95-96	
	GII.4 Yerseke	
	GII.5	87.5 %
	GII.6	87.5 %
	GII.7	87.5 %
	GII.8	87.5 %
	GII.9	87.5 %
	GII.10	87.5 %
	GII.12	90.5%
	GII.13	87.5 %
	GII.14	87.5 %
	GII.16	92.5%
	GII.17	87.5%
	GII.21	87.5%

Figure S2. BLAST results and the threshold selection process for genogroup GI. The selected percentages for each genotype are shown.

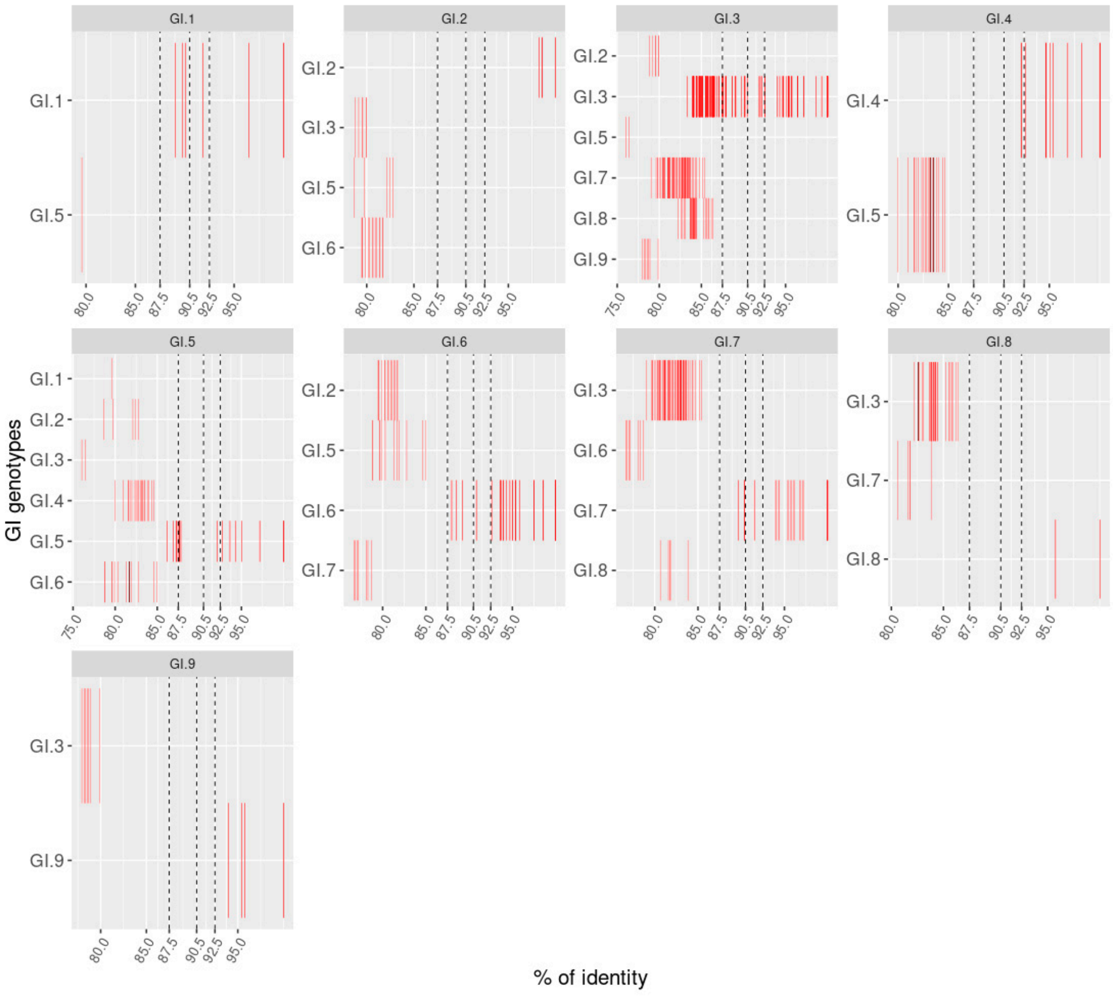
GI.1
GI.2
GI.3
GI.4
GI.5
GI.6
GI.7
GI.8
GI.9
GI genotypes
80.0
85.0
87.5
90.5
92.5
95.0
75.0
% of identity

Figure S3. BLAST results and the threshold selection process for genogroup GII. The selected percentages for each genotype are shown.

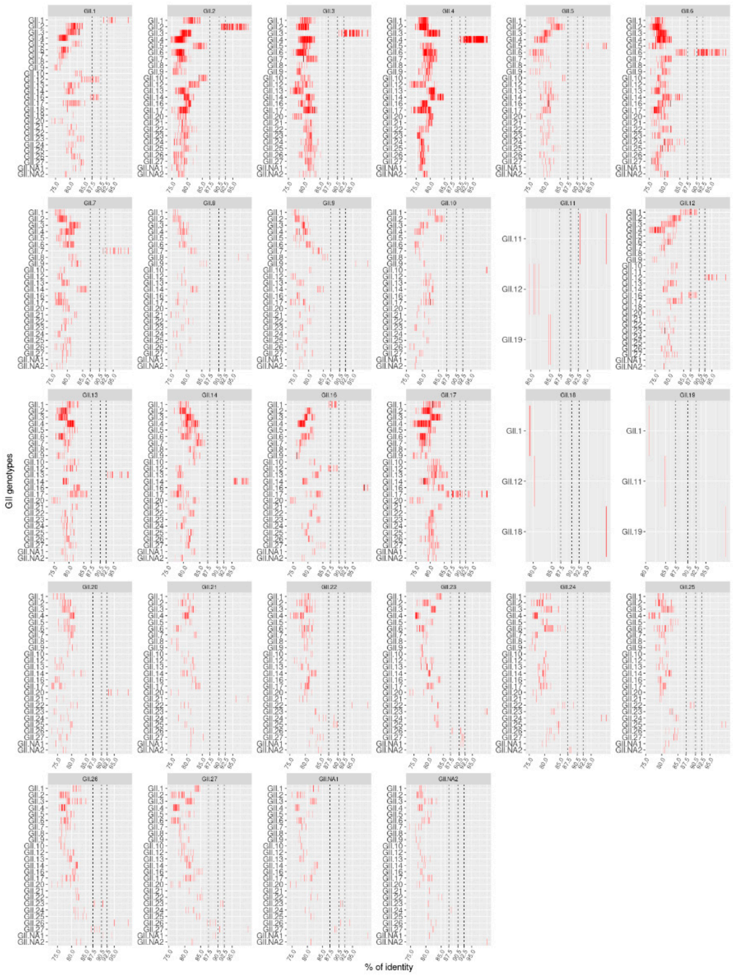

Table S5. Results obteined from RT-nested PCR for NoV-GI (330-bp fragment) and NoV-GII (344-bp fragment).

ID SAMPLE	WWTPs	SAMPLING DATE	NoV-GI	NoV-GII
2472	WWTP1	21/06/2017	-	-
2473	WWTP2	21/06/2017	-	-
2475	WWTP3-A	26/06/2017	-	-
2476	WWTP3-B	26/06/2017	-	-
2500	WWTP1	18/07/2017	-	+
2501	WWTP2	18/07/2017	-	+
2503	WWTP3-A	18/07/2017	+	+
2504	WWTP3-B	18/07/2017	+	+
2506	WWTP3-B	09/08/2017	-	+
2508	WWTP1	09/08/2017	-	+
2509	WWTP2	09/08/2017	-	-
2516	WWTP4	09/09/2017	-	-
2514	WWTP1	19/09/2017	-	-
2515	WWTP2	19/09/2017	-	-
2517	WWTP3-A	19/09/2017	-	+
2518	WWTP3-B	19/09/2017	-	-
2689	WWTP4	15/10/2017	-	+
2690	WWTP1	18/10/2017	-	+
2691	WWTP2	23/10/2017	-	-
2519	WWTP3-A	24/10/2017	+	+
2520	WWTP3-B	24/10/2017	+	+
2545	WWTP4	15/11/2017	-	+

2542	WWTP3-A	21/11/2017	-	+
2543	WWTP3-B	21/11/2017	-	+
2544	WWTP1	24/11/2017	-	+
2550	WWTP4	15/12/2017	+	+
2549	WWTP1	20/12/2017	-	+
2547	WWTP3-A	22/12/2017	-	+
2595	WWTP2	09/01/2018	-	+
2941	WWTP3-A	09/01/2018	+	+
2593	WWTP1	10/01/2018	-	+
2594	WWTP4	15/01/2018	-	+
2591	WWTP3-A	25/01/2018	+	+
2942	WWTP3-B	12/02/2018	+	+
2598	WWTP1	15/02/2018	+	+
2599	WWTP4	15/02/2018	+	+
2600	WWTP2	21/02/2018	+	+
2597	WWTP3-B	22/02/2018	-	+
2604	WWTP4	15/03/2018	+	-
2601	WWTP3-A	21/03/2018	+	+
2603	WWTP1	22/03/2018	+	-
2693	WWTP1	05/04/2018	-	-
2697	WWTP1	05/04/2018	-	-
2692	WWTP4	15/04/2018	-	-
2805	WWTP2	17/04/2018	+	+
2632	WWTP3-A	24/04/2018	+	+
2662	WWTP4	15/05/2018	-	+

2659	WWTP3-A	23/05/2018	+	-
2660	WWTP3-B	23/05/2018	-	+
2661	WWTP1	24/05/2018	-	+
2698	WWTP1	24/05/2018	-	-
2663	WWTP2	28/05/2018	-	+
2802	WWTP4	15/06/2018	+	+
2694	WWTP1	19/06/2018	-	-
2695	WWTP3-A	27/06/2018	-	-
2696	WWTP3-B	27/06/2018	-	-
2707	WWTP3-A	27/07/2018	+	+
2708	WWTP3-B	27/07/2018	+	+
2807	WWTP1	30/07/2018	-	+
2808	WWTP1	01/08/2018	+	+
2798	WWTP2	20/08/2018	-	+
2715	WWTP3-A	31/08/2018	+	-
2716	WWTP3-B	31/08/2018	+	-
2806	WWTP2	01/09/2018	-	+
2809	WWTP1	01/09/2018	+	+
2723	WWTP3-A	30/09/2018	+	+
2724	WWTP3-B	30/09/2018	+	+
2810	WWTP1	01/10/2018	-	+
2743	WWTP3-A	29/10/2018	+	-
2744	WWTP3-B	29/10/2018	+	-
2799	WWTP2	31/10/2018	-	+
2811	WWTP1	01/11/2018	-	+

2775	WWTP3-A	26/11/2018	-	+
2776	WWTP3-B	26/11/2018	-	+
2803	WWTP2	27/11/2018	+	+
2812	WWTP1	01/12/2018	-	+
2777	WWTP3-A	06/12/2018	+	+
2778	WWTP3-B	06/12/2018	+	+
2804	WWTP2	12/12/2018	+	+
2800	WWTP2	28/12/2018	+	+
2813	WWTP1	01/01/2019	-	+
2790	WWTP3-A	30/01/2019	+	+
2791	WWTP3-B	30/01/2019	+	+
2801	WWTP2	30/01/2019	-	-
2868	WWTP3-A	27/02/2019	+	+
2869	WWTP3-B	27/02/2019	+	+
3112	WWTP2	28/02/2019	-	+
3152	WWTP1	01/03/2019	+	+
3113	WWTP2	11/03/2019	+	+
2920	WWTP3-A	27/03/2019	+	+
2921	WWTP3-B	27/03/2019	+	+
3153	WWTP1	01/04/2019	+	+
3119	WWTP2	02/04/2019	+	+
2928	WWTP3-A	24/04/2019	+	+
2929	WWTP3-B	24/04/2019	+	+
3114	WWTP2	24/04/2019	+	+
3154	WWTP1	01/05/2019	+	+

3115	WWTP2	31/05/2019	+	-
3155	WWTP1	01/06/2019	-	+
2982	WWTP3-A	26/06/2019	+	+
2983	WWTP3-B	26/06/2019	+	+
3116	WWTP2	30/06/2019	+	-
3156	WWTP1	01/07/2019	-	+
3117	WWTP2	17/07/2019	-	+
3087	WWTP3-A	30/07/2019	-	+
3088	WWTP3-B	30/07/2019	-	+
3157	WWTP1	01/08/2019	-	+
3110	WWTP3-A	30/08/2019	-	+
3111	WWTP3-B	30/08/2019	-	+
3158	WWTP1	01/09/2019	-	+
3118	WWTP2	17/09/2019	-	+
3134	WWTP3-A	27/09/2019	+	+
3135	WWTP3-B	27/09/2019	+	+
3256	WWTP2	01/01/2020	-	-
3201	WWTP4	16/01/2020	-	-
3203	WWTP3-A	17/01/2020	+	+
3204	WWTP3-B	17/01/2020	+	-
3202	WWTP1	24/01/2020	+	+
3208	WWTP3-A	05/02/2020	+	+
3211	WWTP3-A	10/02/2020	+	+
3213	WWTP4	11/02/2020	-	-
3215	WWTP3-B	11/02/2020	+	-

3384	WWTP1	11/02/2020	-	-
3398	WWTP2	06/04/2020	-	+
3275	WWTP3-A	15/04/2020	-	-
3278	WWTP3-B	16/04/2020	+	-
3445	WWTP3-A	10/06/2020	-	-
3502	WWTP4	20/06/2020	-	-
3487	WWTP3-A	14/07/2020	-	-
3488	WWTP3-B	14/07/2020	-	-
3491	WWTP2	15/07/2020	-	-
3545	WWTP4	16/07/2020	-	-
3550	WWTP1	20/07/2020	-	-
3546	WWTP4	25/07/2020	-	-
3587	WWTP2	05/08/2020	-	-
3593	WWTP3-A	12/08/2020	-	-
3599	WWTP4	14/08/2020	-	-
3591	WWTP1	24/08/2020	-	+
3601	WWTP3-B	02/09/2020	-	-
3700	WWTP2	06/09/2020	-	-
3609	WWTP1	15/09/2020	-	-
3610	WWTP3-A	15/09/2020	-	-
3611	WWTP3-B	15/09/2020	-	-
3697	WWTP4	16/09/2020	-	-
3723	WWTP2	07/10/2020	+	-
3730	WWTP4	13/10/2020	-	+
3716	WWTP3-A	14/10/2020	-	-

3717	WWTP3-B	14/10/2020	-	-
3721	WWTP1	21/10/2020	+	-
3828	WWTP3-A	12/01/2021	-	+
3829	WWTP3-B	12/01/2021	-	-
3825	WWTP2	13/01/2021	-	-
3827	WWTP1	13/01/2021	-	+
3831	WWTP4	13/01/2021	+	+
4004	WWTP3-A	08/02/2021	-	+
4005	WWTP3-B	08/02/2021	-	+
4007	WWTP2	09/02/2021	+	+
4010	WWTP4	11/02/2021	+	+
4013	WWTP1	11/02/2021	-	-
4072	WWTP2	10/03/2021	-	+
4074	WWTP4	10/03/2021	+	+
4070	WWTP1	12/03/2021	-	+
4078	WWTP3-A	12/03/2021	-	+
4079	WWTP3-B	12/03/2021	-	+
4118	WWTP2	07/04/2021	-	-
4113	WWTP3-A	08/04/2021	-	+
4114	WWTP3-B	08/04/2021	-	-
4109	WWTP1	15/04/2021	-	+
4112	WWTP4	15/04/2021	-	+
4190	WWTP3-A	12/05/2021	-	+
4191	WWTP3-B	12/05/2021	-	-
4182	WWTP2	14/05/2021	-	+

4186	WWTP4	14/05/2021	-	+
4185	WWTP1	18/05/2021	+	+
4217	WWTP3-A	11/06/2021	-	-
4218	WWTP3-B	11/06/2021	-	+
4225	WWTP4	11/06/2021	-	+
4222	WWTP2	15/06/2021	-	-
4267	WWTP1	09/07/2021	-	+
4265	WWTP4	13/07/2021	-	+
4266	WWTP2	13/07/2021	-	+
4263	WWTP3-A.	14/07/2021	-	-
4264	WWTP3-B.	14/07/2021	-	+
4280	WWTP1	13/08/2021	+	+
4276	WWTP4	14/08/2021	-	+
4216	WWTP1	16/06/2021	-	-
4284	WWTP3-A.	17/08/2021	+	+
4285	WWTP3-B.	17/08/2021	-	+
4287	WWTP3-B.	24/08/2021	+	-
4278	WWTP2	29/08/2021	-	+
4306	WWTP2	22/09/2021	-	+
4309	WWTP3-A	22/09/2021	+	+
4310	WWTP3-B	22/09/2021	-	+
4307	WWTP1	23/09/2021	+	+
4315	WWTP4	28/09/2021	-	+
4316	WWTP4	01/10/2021	+	+
4308	WWTP1	04/10/2021	-	+

4313	WWTP3-A	06/10/2021	-	+
4314	WWTP3-B	06/10/2021	-	+
4425	WWTP1	03/01/2022	-	-
4327	WWTP3-A	14/01/2022	-	-
4328	WWTP3-B	14/01/2022	-	-
4426	WWTP1	18/01/2022	-	-
4427	WWTP1	21/01/2022	-	-
4428	WWTP1	27/01/2022	-	-
4329	WWTP3-A	15/02/2022	+	+
4330	WWTP3-B	15/02/2022	+	-
4334	WWTP3-A	17/03/2022	+	+
4335	WWTP3-B	17/03/2022	+	+
4766	WWTP4	21/03/2022	+	+
4429	WWTP1	25/03/2022	-	-
4767	WWTP4	01/04/2022	+	+
4337	WWTP3-A	21/04/2022	+	+
4338	WWTP3-B	21/04/2022	+	+
4430	WWTP1	02/05/2022	-	-
4768	WWTP4	03/05/2022	-	+
4417	WWTP3-A	26/05/2022	+	+
4418	WWTP3-B	26/05/2022	+	+
4769	WWTP4	08/06/2022	+	+
4431	WWTP1	10/06/2022	+	-
4451	WWTP3-A	14/06/2022	+	-
4452	WWTP3-B	14/06/2022	+	-

4770	WWTP4	13/07/2022	+	-
4731	WWTP3-A	20/07/2022	-	+
4732	WWTP3-B	20/07/2022	-	+
4771	WWTP4	01/08/2022	+	-
4676	WWTP2	12/08/2022	+	+
4497	WWTP3-A	29/08/2022	+	-
4498	WWTP3-B	29/08/2022	+	-
4679	WWTP1	05/09/2022	+	-
4772	WWTP4	05/09/2022	+	-
4680	WWTP1	13/10/2022	+	+
4511	WWTP3-A	14/10/2022	+	-
4512	WWTP3-B	14/10/2022	+	-
4513	WWTP3-A	31/10/2022	+	+
4514	WWTP3-B	31/10/2022	+	+
5132	WWTP2	20/02/2023	+	+
4761	WWTP3-B	21/02/2023	-	-
4762	RWWTP1	22/02/2023	-	-
4763	WWTP1	22/02/2023	+	+
4764	WWTP4	22/02/2023	+	+
4765	WWTP4	22/02/2023	+	-
4848	WWTP4	17/03/2023	+	+
5133	WWTP2	20/03/2023	+	+
4828	WWTP3-B	29/03/2023	+	+
4847	WWTP1	13/04/2023	+	+
5134	WWTP2	15/04/2023	+	-

4837	WWTP1	18/04/2023	+	+
4839	WWTP4	18/04/2023	+	+
4840	WWTP4	18/04/2023	+	-
4841	WWTP3-A	18/04/2023	+	+
4842	WWTP3-A	18/04/2023	+	+
4843	WWTP3-B	18/04/2023	+	+
4844	WWTP3-B	18/04/2023	+	-
4962	WWTP3-A	08/06/2023	+	+
4965	WWTP3-B	25/06/2023	+	+
4967	WWTP3-A	10/07/2023	+	-
4970	WWTP3-B	10/07/2023	+	-
4976	WWTP1	10/07/2023	+	+
5143	WWTP4	07/08/2023	+	+
5141	WWTP1	14/08/2023	-	-
5074	WWTP3-A	29/08/2023	-	+
5075	WWTP3-B	29/08/2023	-	+
5144	WWTP4	17/09/2023	+	+
5142	WWTP1	18/09/2023	+	-
5145	WWTP3-A	28/09/2023	+	+
5146	WWTP3-B	28/09/2023	+	+
5124	WWTP3-A	17/10/2023	-	-
5125	WWTP3-B	17/10/2023	+	+
5126	WWTP4	17/10/2023	+	+
5127	WWTP1	17/10/2023	-	+
5148	WWTP3-A	22/11/2023	+	+

5149	WWTP3-B	22/11/2023	+	+
5150	WWTP1	22/11/2023	+	+
5151	WWTP3-A	29/11/2023	+	+
5152	WWTP3-B	29/11/2023	-	+
5153	WWTP1	29/11/2023	+	+
5154	WWTP3-A	05/12/2023	+	+
5155	WWTP3-B	05/12/2023	+	+
5156	WWTP1	05/12/2023	-	+
5157	WWTP3-A	13/12/2023	-	+
5162	WWTP3-B	20/12/2023	-	+
5163	WWTP1	28/12/2023	+	+
5164	WWTP3-A	28/12/2023	-	+
5165	WWTP3-B	28/12/2023	+	+
5158	WWTP3-B	13/12/2023	+	+
5159	WWTP1	15/12/2023	+	+
5160	WWTP1	20/12/2023	+	-
5161	WWTP3-A	20/12/2023	+	-
5166	WWTP1	03/01/2024	+	+
5167	WWTP3-A	03/01/2024	+	+
5168	WWTP3-B	03/01/2024	+	+
5169	WWTP1	10/01/2024	+	+
5170	WWTP3-A	10/01/2024	-	+
5171	WWTP3-B	10/01/2024	+	+
5172	WWTP1	17/01/2024	+	+
5173	WWTP3-A	17/01/2024	+	+

5174	WWTP3-B	17/01/2024	+	+
5175	WWTP3-A	23/01/2024	+	+
5177	WWTP3-B	23/01/2024	+	+
5179	WWTP1	23/01/2024	-	+
5214	WWTP3-A	01/02/2024	+	+
5215	WWTP3-B	01/02/2024	+	+
5216	WWTP1	01/02/2024	+	+
5217	WWTP3-A	08/02/2024	+	+
5218	WWTP3-B	08/02/2024	+	+
5219	WWTP1	08/02/2024	+	-
5241	WWTP1	14/02/2024	+	+
5242	WWTP3-A	14/02/2024	+	+
5243	WWTP3-B	14/02/2024	+	+
5274	WWTP4	17/02/2024	-	-
5244	WWTP1	21/02/2024	+	+
5245	WWTP3-A	21/02/2024	-	+
5246	WWTP3-B	21/02/2024	+	+
5247	WWTP3-A	28/02/2024	+	+
5248	WWTP3-B	28/02/2024	+	+
5249	WWTP1	28/02/2024	+	+
5250	WWTP3-A	06/03/2024	+	+
5251	WWTP3-B	06/03/2024	-	+
5252	WWTP1	06/03/2024	+	-
5253	WWTP3-A	13/03/2024	+	+
5254	WWTP3-B	13/03/2024	-	-

5255	WWTP1	13/03/2024	-	-
5256	WWTP1	20/03/2024	+	+
5264	WWTP3-A	20/03/2024	+	+
5265	WWTP3-B	20/03/2024	+	+
5275	WWTP4	27/03/2024	-	-
5266	WWTP3-A	15/04/2024	-	-
5267	WWTP3-A	15/04/2024	-	+
5268	WWTP3-B	15/04/2024	-	-
5269	WWTP3-B	15/04/2024	-	+
5270	WWTP1	15/04/2024	-	-
5271	WWTP1	15/04/2024	-	-
5272	WWTP4	15/04/2024	-	-
5273	WWTP4	15/04/2024	-	-
5298	WWTP3-A	29/05/2024	-	-
5299	WWTP3-A	29/05/2024	+	+
